# Atemporal equilibria: pro- and retroactive coding in the dynamics of cognitive microstructures

**DOI:** 10.3389/fpsyg.2014.00990

**Published:** 2014-09-12

**Authors:** Mark A. Elliott

**Affiliations:** School of Psychology, National University of Ireland GalwayGalway, Ireland

**Keywords:** oscillatory synchronization, gamma band, pro- and retroactive microcognition, protention, visual-event coding

## Abstract

Synchronization of spatially distributed neural assemblies at frequencies in the range 30–70 Hz (the “gamma” band) may be instrumental in grouping stimulus features. In agreement with this we have shown that detection reaction times to a grouping target stimulus are expedited when the stimulus is preceded by repeated presentation of a priming stimulus, presented below detection thresholds in a matrix that flickers at particular frequencies in the 27–68 Hz range. This dynamic priming effect can be partly explained as a function of the return phase of the priming stimulus relative to the premask matrix, indicating one of the primary consequences of repeating stimulation is pre-activation of a priming response relative to prime-stimulus presentation. However, this cannot entirely explain the relationship that develops between the timing of stimulus events (in this instance the time of target relative to priming-stimulus presentations) and response. By varying the frequency and phase of priming-stimulus and target presentations we discovered that given a particular relationship between the phase of target presentation relative to the return phase of the prime, target coding is expedited by a prime that achieves its maximum activation at a phase that would precede priming-stimulus presentation by several tens of milliseconds. However, and in addition, the cognition concerned is flexible enough to be able to achieve an identical prime retroactively, that is to say at a phase during or subsequent to priming-stimulus presentation. This occurs because of a different relationship between the phase of target presentation (defined relative to prime frequency) and the frequency of premask-matrix presentation. On this basis, it can be concluded that by virtue of the relationship between its dynamics and the timing of stimulus events, microstructural cognition functions in a temporal context that can shift from past to future states. Consequently and at the lowest level of psychological function, the conventional, one-dimensional model of time flow—from future to past states does not fully explain how cognition can function. In fact depending upon the interaction in phase between different coding frequencies, the same form of cognition can anticipate or retroactively code events. Consequently, and in so far as our cognition at this level provides a content structure for consciousness, our psychological lives may be fundamentally based upon the ability of our cognitive states to travel backwards and forwards across very short intervals of time.

## Introduction

The rhythmic synchronization of neural activity at gamma-band frequencies (30–70 Hz) is believed to be related to the organization of visual events, in particular the binding of individual visual features to form perceptual wholes (Gray, 1999; Singer, 1999). The precise mechanisms concerned in bringing this about are a matter of discussion, one that has from time to time concerned itself with the relationship between the timing of neural and stimulus or other non-proximal events. For instance, recent focus has involved discussion of the relationship between small eye movements (microsaccades) and induced cortical gamma activity, suggesting that recordings of cortical gamma-band activity are essentially related to the frequency of muscular movements in the eye (Yuval-Greenberg et al., 2008, 2009; Bosman et al., 2009; Melloni et al., 2009a,b; see also Hassler et al., 2011 for contradictory evidence). An older discussion has identified a link between stimulus-evoked gamma activity and visual grouping, suggesting that at least the onset of the gamma response is time locked to a stimulus event (Herrmann et al., 1999; Herrmann and Bosch, 2001; Herrmann and Mecklinger, 2001, see Tallon-Baudry, 2009; Martinovic and Busch, 2011 for reviews). This is a controversial theory as other EEG, as well as the physiological literature usually reports oscillatory-gamma activity to be unrelated to the phase of stimulus events (see Tallon-Baudry and Bertrand, 1999 as well as Pantev, 1995; Fries et al., 2007, for reviews).

**Figure 1:**
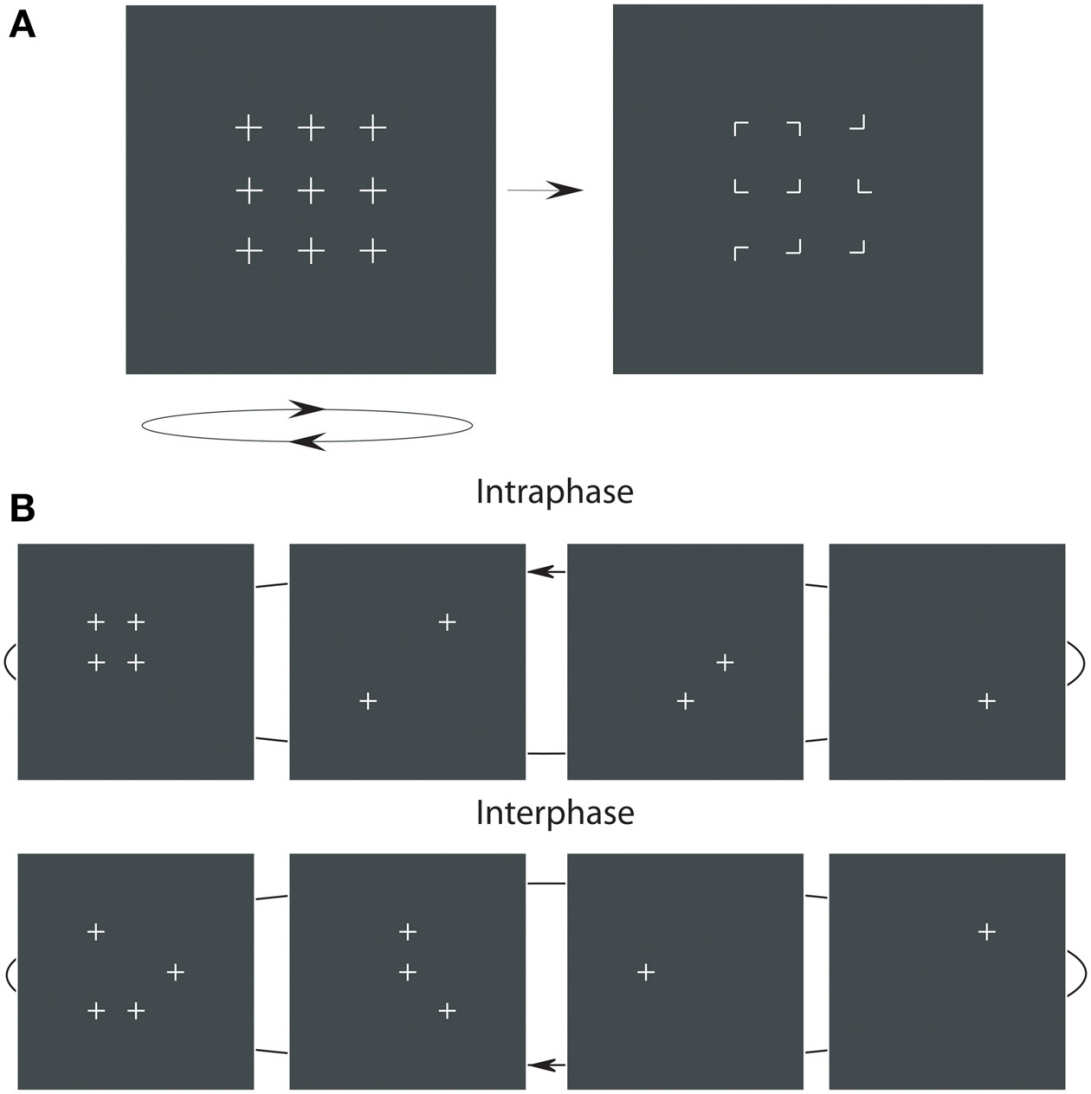
**The premask-matrix presentation paradigm**. In **(A)** upon termination the oscillating premask-matrix was immediately followed by presentation of a target display comprising 90°-corner junctions to which observers had to make a speeded target (i.e., Kanizsa square) present or, if not presented, an absent response. In **(B)** are shown example sequences of four image frames that comprise the premask matrix. In the intraphase conditions (upper panels) one premask frame consists of four crosses in square arrangement; see the upper far left panel. In the interphase condition (lower panels) these four crosses are distributed across frames.

There are also a number of psychophysical studies of the relationships between stimulus timing and the synchronized appearance of stimulus elements, feature binding and visual grouping. Of these, and taking into account critiques that challenge the necessity and sufficiency of some synchronization paradigms (Farid, 2002; Elliott et al., 2006b), there are a class of paradigms that have employed stimulus synchronizations presented below detection thresholds which prime or bring about the Gestalt organization of the synchronized stimulus elements (Elliott and Müller, 1998; Usher and Donnelly, 1998). These studies acknowledge a now large body of evidence indicating that elements of a visual scene are often bound ahead of attentional deployment and are very unlikely to be coded by the same mechanisms as those mediating direct conscious experience of the Gestalt (e.g., Duncan and Humphreys, 1989; Rensink and Enns, 1995; Driver et al., 2001). Usher and Donnelly showed that synchronization of orientation at a frequency in the gamma band significantly biases subsequent orientation judgments even though observers cannot reliably report the synchronized orientation. In Elliott and Müller's paradigm (Figure 1), embedding a figurally-relevant grouping as one phase of a multiphase premask that flickered at 40 Hz, leads to faster detection reaction times (RTs) to a subsequently presented target grouping, without prior attentional deployment to the location of the grouping in the premask. Observers could not detect the presence of the premask grouping and because presentation of this stimulus does not cue target presentation, it is referred to as a prime.

**Figure 2:**
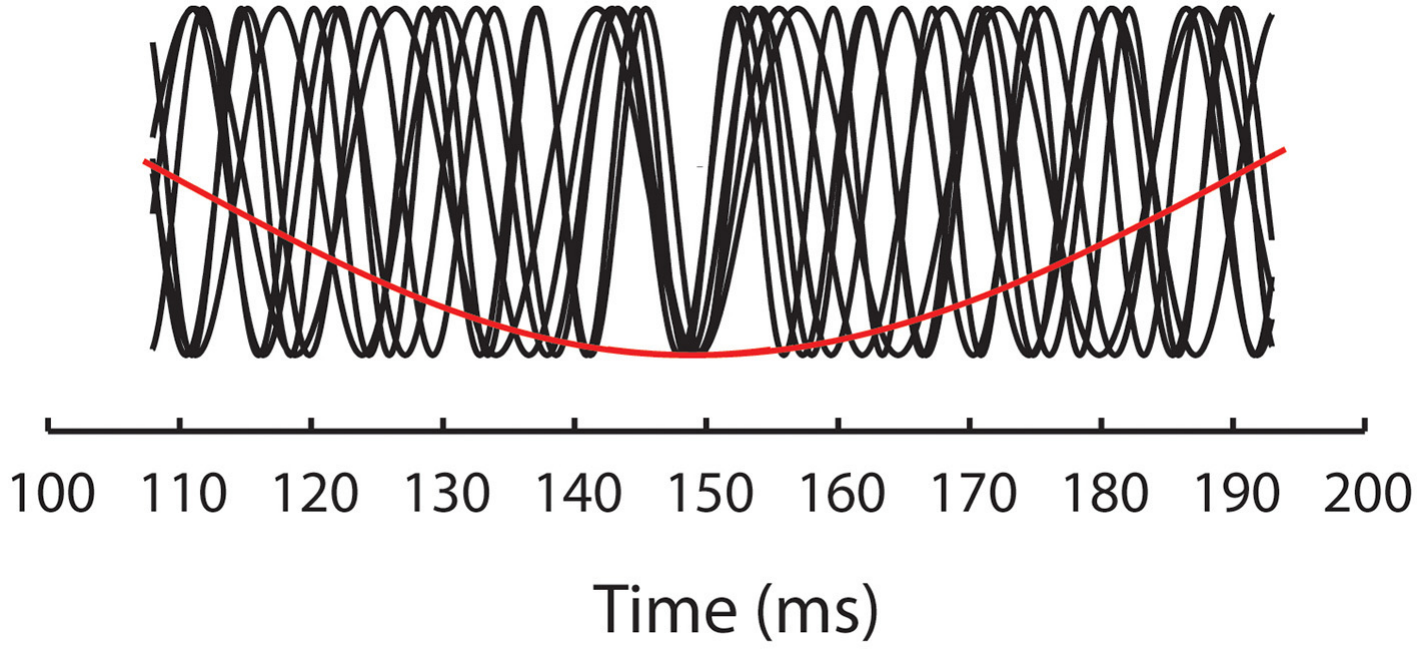
**Illustrates the GPAH model of oscillatory priming in which the priming frequencies (33, 39–40, and 46–47 Hz—black functions) align phases at 148 ms and are thus also phase aligned with a stimulus-evoked rhythm of 6.75 Hz (red function)**.

What is surprising about the results reported by Elliott and Müller (1998) is the specificity of the priming effects to 40 Hz. Elliott and Müller (2004) subsequently discussed a set of experiments in which premask matrices were presented at frequencies in single Hertz steps over the range 30–50 Hz. These experiments extended upon the original finding reported by Elliott and Müller (1998) in that they showed priming effects not to be confined to 40 Hz. Instead, priming was found when premask matrices flickered at 33 Hz, 39–40 Hz and at 46–47 Hz. Described in terms of a “Generalized Phase Angle Hypothesis” (GPAH), Elliott and Müller (2004) observed that priming occurs for primes presented within premask rhythms that would all be in phase alignment at regular 148 ms intervals, implicating modulation of the premask-presentation rhythm by a slower (EEG theta) rhythm of approximately 6.75 Hz with which they would share a common phase angle (see Figure 2). The GPAH predicts that, for a given priming frequency *f* and corresponding period duration τ = 1/*f*, facilitation reoccurs at every time point.

J(τ)=(nτ+ ½)•τ−T

where *n*τ is a frequency-specific integer multiplier and *T* denotes a constant quantal time delay. The term +½ accounts for the observation that, for *f* = 40 Hz, maximal facilitation occurs at phase angles of 180° relative to the rhythm of premask-matrix presentation (Elliott and Müller, 2000).

Prime generation given regularly ordered stimulus frequencies in phase with a slower, presumably endogenous rhythm is only one part of the story. An analysis of the same dataset, published earlier by Kompass and Elliott (2001) found that priming varied in magnitude (or priming was or was-not present) for frequencies according to the time of target presentation expressed in terms of the phase of the premask-matrix presentation frequency (referred to in terms of a “Return Phase Hypothesis” or RPH for oscillatory priming). In fact priming was maximal for targets presented at a time ahead of the premask-matrix presentation phase at which the priming-stimulus would have been presented if premask-matrix presentation had continued. This indicates the prime to be a cognitive response that can develop *in advance* of the priming stimulus, most likely as a function of the rhythmic nature of premask-matrix presentation.

The picture thus far developed identifies a form of pre-activation of neurons coding the priming stimulus as a function of an interaction between one or more phases of the premask-presentation rhythm and an inferred, but not directly observed rhythm of around 6.75 Hz. This picture is however complicated by the absence of a corresponding EEG response matching the 40-Hz rhythm of premask presentation (Elliott et al., 2000), but the presence nevertheless of a 33–34 Hz response (a rhythm predicted by the GPAH to encourage prime formation, see Elliott et al., 2003). Given that prime formation is preattentive and based upon a stimulus that is not detected (Elliott and Müller, 1998; Shi and Elliott, 2007), while it is not possible to refer to prime generation as “protentive” after Husserl's (1928) definition (which directly concerns an “experienced” future state), it seems that the prime can, under some circumstances, represent the temporal advancement (or pre-activation) of visual cognition. The question explored in this paper is what those circumstances are with the expectation that they are describable in terms of patterns of interaction in the dynamic systems coding the prime.

The work presented here describes the results of 7 experiments in Study 1 followed by 2 subsequent experiments in Study 2. The experiments presented in Study 1 define a very precise temporal relationship between stimulus frequency and phase referenced to a slower endogenous rhythm of 6.69 Hz. It is this relationship that results in maximum prime activation ahead of priming-stimulus presentation. In other words, when coupled with the slow 6.69-Hz rhythm, the frequency of premask-frame presentations preactivates a prime relative to priming-stimulus presentation.

Study 2 extends upon these results and show, by varying premask-matrix presentation time, that primes not only predate but can achieve maxima in synchrony with the presentation phase of the priming stimulus (Experiments 8 and 9). They may also slightly lag the priming-stimulus phase (Experiment 9). These experiments show that prime maxima that predate priming-stimulus presentation rely upon an interaction of slow theta with the gamma-band premask-matrix presentation rhythms—as predicted by the GPAH. Conversely phase aligned and retroactive priming is not based upon this interaction. These may also occur for any frequency depending upon their return phase—as predicted by the RPH. Taken together, these experiments show the temporal relations required for prime pre-activation as well as for retroactive priming, allowing us to discuss the role dynamic microcognition plays in defining a cognitive equilibrium (a state in which the immediate outcome of cognition is stable and successful) that extends across a small interval spanning future and past time.

## Study 1 materials and methods

### Participants

Participant data are given in Table 1. All participants in all experiments had normal or corrected-to-normal vision. They performed one block of practice trials immediately before the experiment proper and were naive as to the precise experimental conditions presented in the experiment. Participants were paid at a rate of 10.00 DM (deutschemark) per hour.

**Table 1:** **Methods Details for Experiments 1–7**.

**#E**	**#*N***	**Mean age (*SD*)**	***f***	**Steps**	**#Trials**	**#Blocks**	**#Sessions**
1	14	22.2 (4.5)	36–43	1	1280	16	2
2a	15	22.5 (5.5)	36–43	1	1280	16	2
2b	15	22.5 (5.5)	44–51	1	1280	16	2
2c	15	22.5 (5.5)	28–35	1	1280	16	2
3	12	24.8 (2.4)	54–65	1	1920	16	3
4a	12	23.4 (3.2)	27.75–29.5	0.25	1280	16	2
4b	12	23.4 (3.2)	32.5–34	0.25	1280	16	2
4c	12	23.4 (3.2)	45.25–48	0.25	2304	16	3
5	11	24.1 (3.4)	53.25–55.5	0.25	1600	20	2
6	11	23.3 (4.3)	59–60.75	0.25	1280	16	2
7	11	23.3 (4.3)	65.75–67.5	0.25	1280	16	2

*#E refers to experiment number; #N to number of participants; Mean age and (SD) standard deviation in years; f to the premask-matrix presentation frequency range (in Hertz); Steps to the resolution (in Hertz) between steps; #Trials to the number of trials per participant; #Blocks to the number of blocks per session; #Sessions to the number of experimental sessions. E#2a–c and also E#4a–c were run using the same participants, within experiments, and with counterbalanced experimental order*.

All participants provided informed consent to participate in the experiments, with protocols approved by departmental research ethics committees convened at the University of Leipzig and the Ludwig-Maximilians University, Munich.

### Apparatus and stimuli

Stimulus image frame generation, event timing, and data collection were controlled by an IBM compatible PC, which also controlled oscilloscopic image presentation through an Interactive Electronics Systems point plotter buffer with 8 Mb frame store memory (Finley, 1985). Image frames were presented on a 6″ Tektronix 608-oscilloscope monitor equipped with a very fast-decay P15 phosphor. The use of a P15 phosphor ensured that on-screen image persistence reduced to 10% of normal image intensity within 2.8 μs (microseconds) of image termination (Bell, 1970). The Interactive Electronic Systems point plotter buffer allowed pixels to be plotted at a rate of one pixel every microsecond.

The stimulus display (illustrated in Figure 1) consisted of a matrix of premask elements distributed across 4 sequentially presented premask-matrix image frames. In Experiments 1–7, this sequence of image frames was presented rapidly and repeatedly for 600 ms, after which the premask matrix transformed into a static target matrix of corner junctions. Under target conditions a subset of these junctions would group in one display region to form an illusory target square (illustrated in Figure 1A right). Participants viewed these displays at a distance of 57 cm, maintained via a chin rest. Experiments were conducted in a dimly lit room (mean screen surround luminance 0.078 cd/m^2^), with stimulus luminance maintained at 0.3 cd/m^2^upon a background field of 0.075 cd/m^2^. The 3 × 3 premask matrix (Figure 1A left), subtended 7°51′ × 7°51′ of visual angle at the center of the screen. Premask elements were crosses of size 51′ and were separated from their nearest horizontal and vertical neighbors by 2°39′. Premask crosses consisted of 19 tightly spaced points, so that their segments appeared as uninterrupted lines. Premask image frames could consist of 1, 2, 3, or 4 crosses presented simultaneously (Figure 1B), so that the number of pixels presented in a given frame were 21, 42, 63, or 84. In order to ensure all frames were equiluminant, an additional 979, 958, 937, or 916 pixels, respectively, were plotted to an invisible corner of the display (with X,Y coordinates 0,0), equalizing the number of pixels plotted in a single frame. Junction elements in the target matrix (Figure 1A right) subtended 26′ of visual angle and were separated horizontally and vertically by between 2°39′ and 3°30′. Each target junction consisted of 11 tightly spaced points, and the target matrix overall consisted of 99 pixels. According to an identical procedure to that used for the premask matrices, an additional 901 pixels were plotted to an invisible corner of the plotter screen. The size and separation of the inducer elements produced a Kanizsa-type square, which, according to recent formulations (Shipley and Kelman, 1992), represents a “good square” with a probability of less than 0.1.

### Design and procedure

Experiments 1–7 were conducted to examine the relationship between priming and the frequency of premask-matrix frame presentations. Accordingly, premask-matrix frequencies were varied and the ranges of frequencies for each experiment are given in Table 1. In Table 1 experiments are listed in running order and followed the logic that a range of frequencies was examined for frequencies of relevance to priming (Experiments 1–3) followed by experiments with higher frequency resolution that provided a more specific estimate of the frequency bandwidths at which primes are generated (Experiments 4–7). Experiments 2a–c examined an additional hypothesis that priming occurs at particular frequencies, but that these may differ across participants. Accordingly these experiments examined the frequency ranges 28–35 Hz, 36–43 Hz, and 44–51 Hz with counterbalanced experimental order. No clear evidence was found to support this idea. Experiments 4a–c also ran with the same participants in counterbalanced order.

Premask-presentation frequency was defined in terms of a constant presentation time for each of the 4 individual premask-image frames. For example, for a premask presentation frequency of 40 Hz the premask-image frames were presented at a rate of 10 repeats per second, which given a constant exposure duration of 25 ms (ms) and an inter-frame interval of less than 1 ms resulted in a matrix frequency of 40 Hz. As illustrated in Figure 1B, premask-matrix presentation was divided into two critical conditions: in the first and on 50% of trials, the premask elements were pseudo-randomly (or “interphasically”) distributed across all 4 image frames with 4 elements appearing in frame 1 controlled to avoid the possibility of accidental, figurally relevant spatial organizations arising within this image frame. This is illustrated in the lower panel of Figure 1B. On the remaining trials, premask-matrix frame 1 included the synchronous (or “intraphase”) presentation of 4 premask elements in square arrangement at the precise matrix locations which could (on 50% of trials) be occupied by the 4 corner junctions defining a Kanizsa-type target square (the target present condition). This is illustrated in the upper panel of Figure 1B. “Kanizsa-type” here refers to the illusory geometrical forms that emerge as a function of the collinear organization of appropriately oriented corner junctions. An example of this is illustrated in Figure 1A (right panel). In a further 50% of trials the matrix of corner junctions presented after premask-matrix presentation included elements that did not group to form an illusory square (the target-absent condition).

The factors in all experiments were Prime (Intra-phase/Inter-phase premask presentation), Target (Present/Absent) and premask-matrix presentation Frequency (*f*—see Table 2). Following a brief computer-generated tone, participants were presented with the flickering 3 × 3 premask matrix which after 600 ms reduced to a matrix of simple 90° corner junctions by removal of superfluous line segments. Participants had then to discern the presence or absence of a Kanizsa-type square (target) within this matrix and produce a target-present/absent reaction time (RT), using one of two separate response keys, as rapidly and accurately as possible. In each experiment all factors were varied randomly: Table 1 details the numbers of blocks, trials and sessions over which participants completed the experiments.

**Table 2:** **%Errors**.

**#**	***f***	**#Trials**	**#Errors**	**%Errors**
1	36–43	17920	586	3.3
2a	36–43	19200	545	2.8
2b	44–51	19200	653	3.4
2c	28–35	19200	565	2.9
3	54–65	23040	209	1.0
4a	27.75–29.5	15360	616	4.0
4b	32.5–34	15360	446	2.9
4c	45.25–48	27648	707	2.6
5	53.25–55.5	17600	351	2.0
6	59–60.75	14080	346	2.5
7	65.75–67.5	14080	363	2.6

*#Refers to experiment number; f to the premask-matrix presentation frequency range (in Hertz); #Trials to the number of trials overall; #Errors to the number of errors; %Errors to the number of errors expressed as a percentage of overall number of trials*.

## Results of study 1

Those trials with error responses (see Table 2) were removed from the data prior to subsequent analyses. Error RTs tended to be slower overall than correct RTs, and analysis of the probability correct by RT revealed no significant correlation between RT and accuracy, which argues against the correct data being contaminated by accuracy-speed trade-offs. Examination of the correct RTs revealed non-normal distribution with pronounced positive skew. A Kolmogorov “D” test showed RT distributions to be approximately lognormal and on this basis subsequent analyses were conducted on the exponents of the means of log-transformed RT distributions (for supporting ideas see Box and Cox, 1964, 1982).

In this study we sought (i) to establish evidence for priming across a wider range of frequencies than those presented by Elliott and Müller (2004), while (ii) better specifying the bandwidths at which priming is discovered. Evidence of priming is typically indicated by significant Target × Prime interaction in the analysis of variance (ANOVA see all experiments reported in Elliott and Müller, 1998, 2000, 2001; Elliott et al., 2000; Conci et al., 2004; Becker et al., 2005; Elliott et al., 2006a; Shi and Elliott, 2007). Resolution of this interaction always refers to a target-specific RT advantage for the intra- vs. interphase premask conditions. Given each experiment examined the factors Target (present/absent and indicative of search), Prime (intra vs. inter and indicative of priming) and *f* (premask-matrix presentation frequency), we sought to resolve either the three way interaction, which would indicate both target- and frequency-specific priming, and/or one or both two-way interactions (Target × Prime; Prime × *f*), which would be expected to indicate target- and frequency-specific priming, in the latter case if the error term was sufficiently large to render the three-way interaction non-significant (perhaps the case given the quite high resolution but narrow frequency bands examined in some experiments). Table 3 presents relevant results of repeated measures ANOVAs carried out for each experiment.

**Table 3:** **RT data ANOVA Table**.

**#**	***f***	***T***	***P***	***T × P***	***P × f***	***T × P × f***
1	36–43	*F*_(1, 13)_ = 57; *p* < 0.001	NS	*F*_(1, 13)_ = 8.4; *p* < 0.025	*F*_(5.6, 73)_ = 2.4; *p* < 0.05	*F*_(5, 65)_ = 2.8; *p* = 0.025
2a–c	28–51	*F*_(1, 14)_ = 53; *p* < 0.001	*F*_(1, 14)_ = 7.4; *p* < 0.025	*F*_(1, 14)_ = 4.5; *p* = 0.051	*F*_(16.6, 232.8)_ = 1.3; *p* < 0.005	NS
3	54–65	*F*_(1, 11)_ = 9.8; *p* = 0.01	NS	*F*_(1, 11)_ = 5.8; *p* < 0.05	NS	NS
4a	27.75–29.5	*F*_(1, 11)_ = 4.5; *p* = 0.059	NS	NS	NS	*F*_(7, 77)_ = 2.1; *p* = 0.053
4b	32.5–34	NS	*F*_(1, 11)_ = 26.7; *p* < 0.001	*F*_(1, 11)_ = 15.6; *p* < 0.005	*F*_(5.4, 59.6)_ = 3.4; *p* < 0.01	NS
4c	45.25–48	*F*_(1, 11)_ = 4.1; *p* = 0.068	*F*_(1, 11)_ = 22.2; *p* < 0.001	*F*_(1, 11)_ = 15; *p* < 0.005	*F*_(7.9, 86.9)_ = 2.1; *p* = 0.05	*F*_(8.8, 96)_ = 2.4; *p* < 0.025
5	53.25–55.5	*F*_(1, 10)_ = 15.3; *p* < 0.005	NS	*F*_(1, 10)_ = 4.5; *p* < 0.06	*F*_(8.6, 86)_ = 2.9; *p* = 0.005	NS
6	59–60.75	*F*_(1, 10)_ = 7.9; *p* < 0.025	*F*_(1, 10)_ = 32.3; *p* < 0.001	*F*_(1, 10)_ = 5.7; *p* < 0.05	NS	NS
7	65.75–67.5	*F*_(1, 10)_ = 23.5; *p* = 0.001	*F*_(1, 10)_ = 10.1; *p* = 0.01	*F*_(1, 10)_ = 4.2; *p* = 0.066	*F*_(6.4, 64.3)_ = 3; *p* < 0.01	NS

*T, Target; P, Prime; f, premask-matrix presentation frequency. Huynh-Feldt or Greenhouse Geisser adjustments applied where sphericity assumptions are not met (see Huynh and Feldt, 1976)*.

With respects to previous studies, the only anomalies are an absence of a target effect in Experiment 4c (indicating absent search to be as efficient as target search) and an inability to resolve the Target × Prime interaction in Experiment 4a. This may be due to the proximity of the examined frequency band to the intraphase-premask detection threshold at 21 Hz: leading to effects on target-absent trials. Note that analysis of the arcsine-transformed error data using repeated-measures ANOVA with the same terms as those used for analysis of the RT data showed no systematic effects (Target or Target × Prime) between experiments, indicating that participants properly performed the target detection task and were not differentially influenced by any frequency or frequency band in doing so. Using the method proposed by Grice et al. (1977), we were unable to find the data of any participant to be influenced by speed-accuracy trade-offs.

Simple-effects analyses were carried out to determine at which frequencies priming occurs using the error terms from the three-way interaction, or from the Prime × *f* interaction resolved in analysis of the target trials only (predicted from a significant Target × Prime interaction carried out for Experiment 3). Priming frequencies are given in Table 4.

**Table 4:** **Priming frequencies**.

**#**	***f***	***P × f***	***Pf***
1	36–43	*F*_(2.9, 37.7)_ = 4.3; *p* < 0.025	{38 Hz, 15 ms; *p* < 0.025} {39 Hz, 30 ms; *p* = 0.001} {40 Hz, 21 ms; *p* < 0.025}
2a–c	28–51		{33 Hz, 22 ms; *p* < 0.025} {38 Hz, 20 ms; *p* < 0.05} {39 Hz, 18 ms; *p* < 0.05} {40 Hz, 21 ms; *p* < 0.025} {46 Hz, 29 ms; *p* = 0.001} {47 Hz, 19 ms; *p* < 0.05}
3	54–65	*F*_(6.2, 67.7)_ = 1.8; *p* = 0.053	{60 Hz, 17 ms; *p* = 0.025}
4a	27.75–29.5		{29.5 Hz, 10 ms; *p* < 0.05}
4b	32.5–34	*F*_(6.7, 74.1)_ = 3.8; *p* < 0.005	{32.25 Hz, 20 ms; *p* < 0.005} {32.5 Hz, 19 ms; *p* = 0.0001} {32.75 Hz, 24 ms; *p* = 0.0001} {33 Hz, 19 ms; *p* = 0.001} {33.25 Hz, 19 ms; *p* = 0.001} {33.5 Hz, 11 ms; *p* < 0.025}
4c	45.25–48		{45.75 Hz, 16 ms; *p* < 0.05} {46 Hz, 22 ms; *p* < 0.01} {46.25 Hz, 21 ms; *p* < 0.025} {46.5 Hz, 22 ms; *p* < 0.01}
5	53.25–55.5	*F*_(8.6, 85.6)_ = 3.3; *p* < 0.005	{53.25 Hz, 20 ms; *p* = 0.01} {53.5 Hz, 22 ms; *p* < 0.01} {53.75 Hz, 20 ms; *p* < 0.05}
6	59–60.75	NS	{59 Hz, 21 ms} {59.25 Hz, 14 ms} {59.5 Hz, 14 ms} {60.25 Hz, 20 ms} {60.5 Hz, 13 ms}
7	65.75–67.5	NS	{65.75 Hz, 20 ms} {66 Hz, 23 ms} {66.5 Hz, 18 ms} {66.75 Hz, 24 ms}

*Prime × f interactions reported here refer to analysis of target trials only. Where not specified the interaction term derives from the omnibus ANOVA reported in Table 3; Reported are prime(f), the magnitude of the priming effects [in milliseconds (ms)] and significance (p). Huynh-Feldt or Greenhouse Geisser adjustments applied where sphericity assumptions are not met. In #6 and #7 a number of frequencies primed resulting in a strong priming main effects [F_(1, 10)_ = 32.9, p < 0.0001 for #6; F_(1, 10)_ = 25.3, p = 0.001 for #7] but with reduced power in the Prime × f interactions. Reported priming frequencies were tested for significance using simple-effects analysis and the error term in the Prime × f interaction. However, in these instances, as the significance values are themselves at best indicative, they are not reported here*.

**Figure 3:**
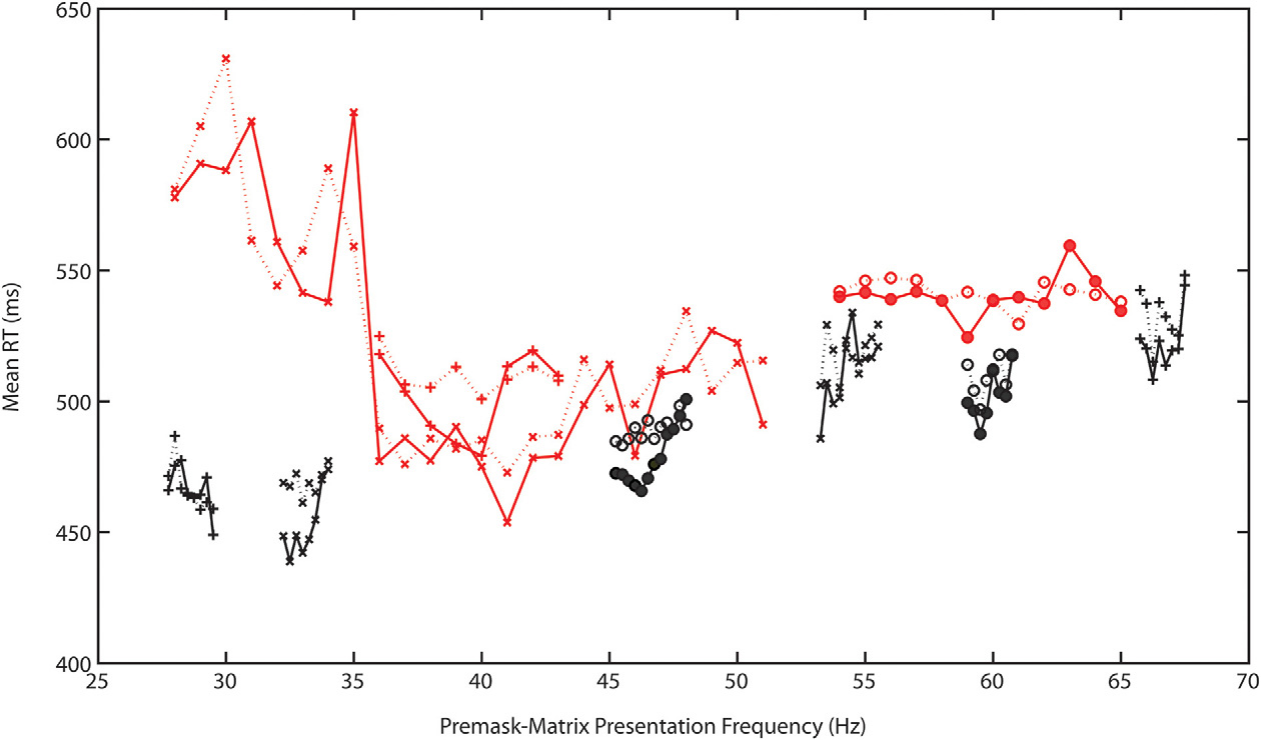
**Target RT data for Experiments 1–7, following removal of a linear slope that describes the general increase in RT with increasing frequency**. Solid lines represent intra- and dashed lines the interphase RTs. Different symbols represent data from different experiments. The red lines indicate the single-Hertz step experiments (#1–3) that typically cover a wider frequency range than the black graphed 0.25-Hz stepped experiments (#4–7).

Preliminary analysis quite clearly shows that the modulated pattern of frequency-specific priming identified and reported by Elliott and Müller (2004) not only replicates across experiments, it may replicate and be specified with a finer resolution across a range spanning (at least) 29.5–67.75 Hz. That is to say across a very major part of the EEG gamma bandwidth. Study 1 shows some evidence for priming at 29.5 Hz alongside more robust evidence at bands spanning 32.25–33.5 Hz; 38–40 Hz; 45.75–46.5 Hz; 53.25–53.75 Hz; with wider bands identified as spanning 59–60.75 and 65.75–67.5 Hz. Although not supported by significant Prime × *f* interactions it is likely that even within these fast-frequency bands there are particular frequencies that prime more efficiently than others. No other frequency tested was associated with priming. Together with the frequency specificity of priming this reinforces the idea that prime formation is a function of the Generalized Phase Angle Hypothesis (GPAH). For illustrative purposes the target RT data for Experiments 1–7, following removal of a linear slope that describes a general increase in RT with increasing frequency, are presented in Figure 3.

**Figure 4:**
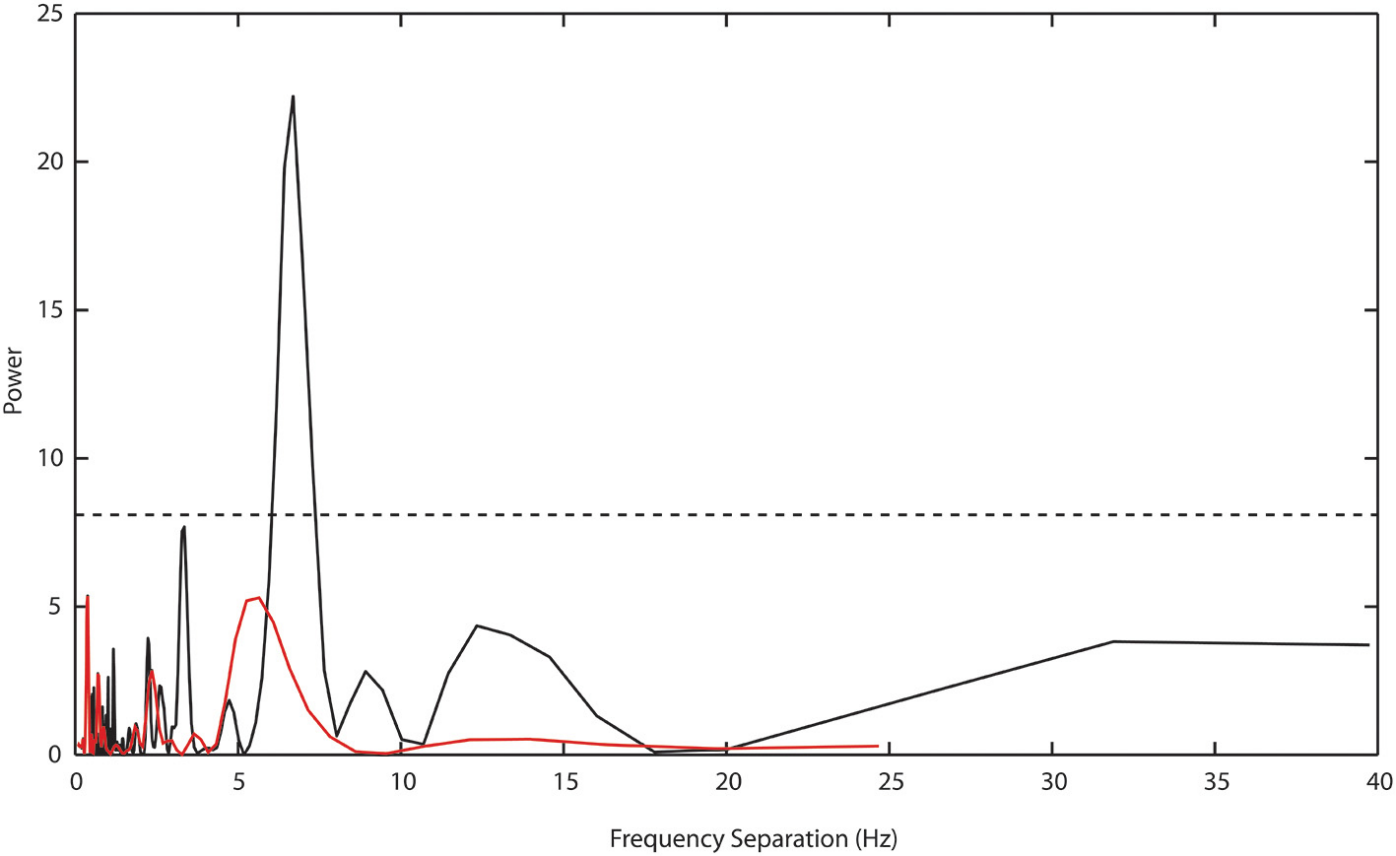
**Lomb-Scargle periodogram describing periodicities in the separation of intra and interphase target RTs across all experiments in Study 1**. A bootstrap estimate of significance at *p* ≤ 0.05 (dashed horizontal line) is set at power values of ≥7.5. A single highly significant peak separation is evident at 6.69 Hz (black function). The red function shows the power estimate derived from the analysis of Experiment 8 data, for which there is no significant peak separation evident at any frequency.

To test the GPAH the Prime × *f* pattern was examined using the Lomb-Scargle or least-squares spectral-analysis method. This method allows for an analysis of cyclical structure with a better resolution than conventional Fourier methods and is designed for short time series or time series with unequal or missing data (Scargle, 1982). Frequency separation between priming bands was originally suggested to be 6.75 Hz. The Lomb-Scargle method was applied to all differences between intra and interphase target RTs over all premask-matrix presentation frequencies in Experiments 1–7, that is to say across both frequencies at which priming was recorded as well as frequencies at which there was no priming (see Table 4). Figure 4, presents the resulting Lomb-Scargle periodogram which exhibits a single significant peak located at 6.69 Hz. This is remarkably close to the original estimate of 6.75 Hz and makes a much more substantive case for consideration of the prime as a function of the interaction in phase between premask-matrix and EEG-theta rhythms at (1000/6.69 =) 149 ms intervals.

Having established that approximately the same frequency separation predicted by the GPAH over the 28–51-Hz range, characterizes priming between approximately 27 and 68 Hz, it is logical to suppose that the data support the Return Phase Hypothesis (RPH). Simple priming (e.g., that shown by Elliott and Müller, 1998, 2000, etc.) usually occurs when the prime is presented immediately prior to target presentation. However, and exploiting the logic that the premask frame (phase of premask presentation) will differ if premask-matrix presentation frequencies differ but presentation time overall remains constant, Kompass and Elliott (2001) concluded that the relative position of frame 1 (containing the priming stimulus) at termination of premask-matrix presentation determines whether it is an effective prime or not. If premask-matrix frame 1 is about to be presented or has just been presented, primed target RTs are maximally reduced leading to larger priming effects.

**Figure 5:**
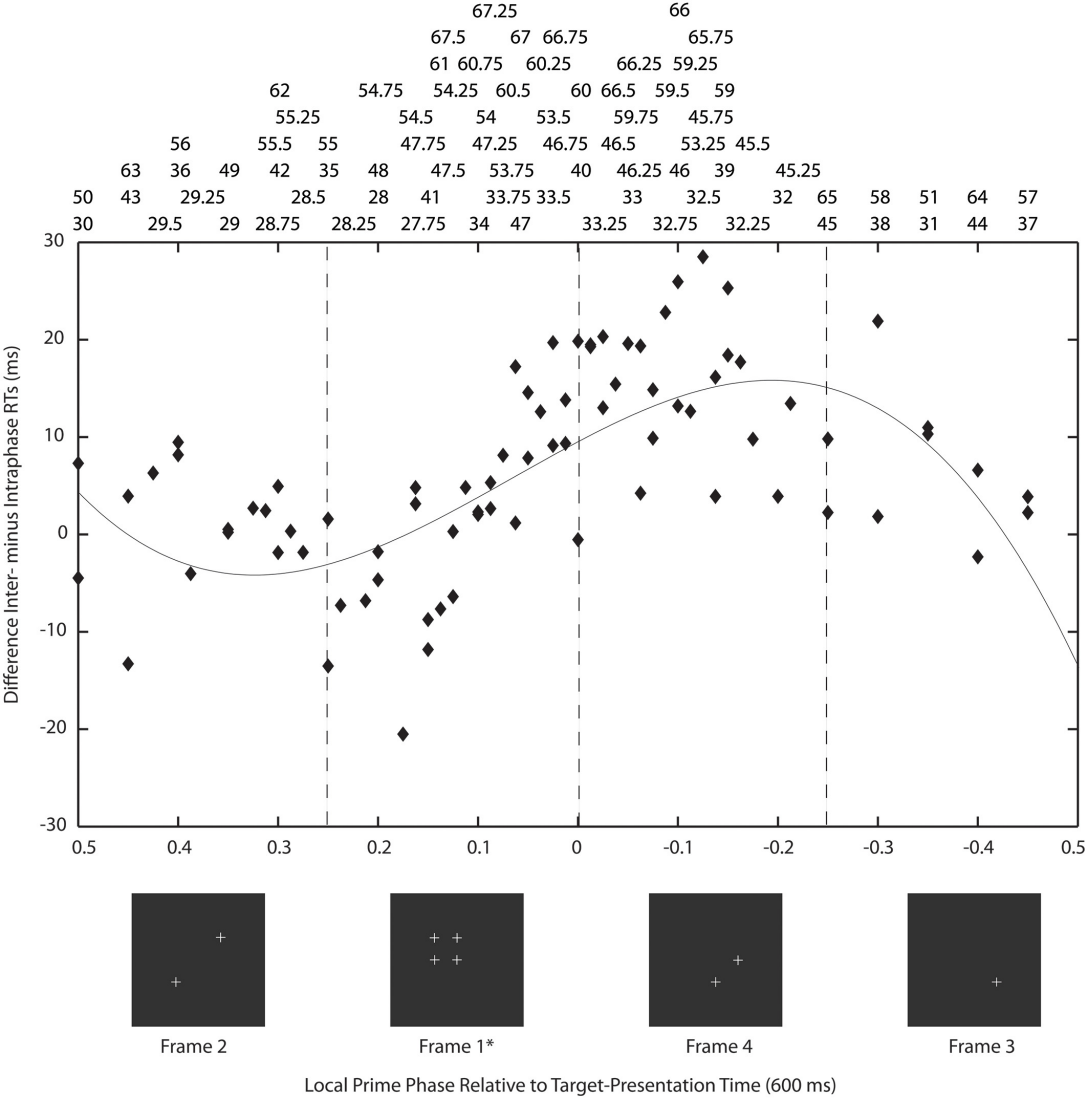
**Priming as a function of return phase: on the ordinate the subtraction of the mean intra- from interphase RTs (for some frequencies the priming effects) are plotted for each frequency over the time of target presentation (600 ms post-trial onset) transformed into a corresponding phase of the premask-matrix cycle**. Here, the abscissa is divided into two where Phase = 0 represents the onset of frame 1—the priming frame (asterisked). To the left of this are represented frequencies at which the target appears at a phase corresponding to frames 1 and 2 and to the right are frequencies at which the target appears at phases corresponding to presentation of frames 3 and 4. Frames are illustrated below in sequence from frame 1 (the priming frame) to the left to frame 2 at which point the sequence continues to the far right proceeding leftwards from frame 3. Frame presentation is cyclical until premask-matrix termination at 600 ms (see Figure 1). Above are listed the frequencies that correspond to data points in the chart. Almost all priming frequencies lie in the phase range 0.025 (53 Hz) to –0.3 (38 Hz) with the very large majority at times corresponding to target presentation in phase with frame 4, that is to say, ahead of priming stimulus (frame 1) presentation. The non-linear function describes the pattern of mean intra- from interphase RTs over premask-matrix phase (for some frequencies this is the magnitude of priming) [*y* = 36 ^*^x^3^ − 14^*^x^2^ − 27^*^x + 11; F_(3,79)_ = 6.23, *p* = 0.001] and shows peak priming to occur at phase −0.193, which collapsed across frequencies is equivalent to approximately ¼ (22.8%) the duration of a single frame (the relevant frame being frame 4). Considering the lowest and least frame-1 proximal of the priming frequencies contributing to the frame 4 peak (32 Hz Table 4), this indicates a fully developed prime as early as 24 ms [(1000/32.5)^*^0.772] ahead of priming-stimulus presentation.

**Figure 6:**
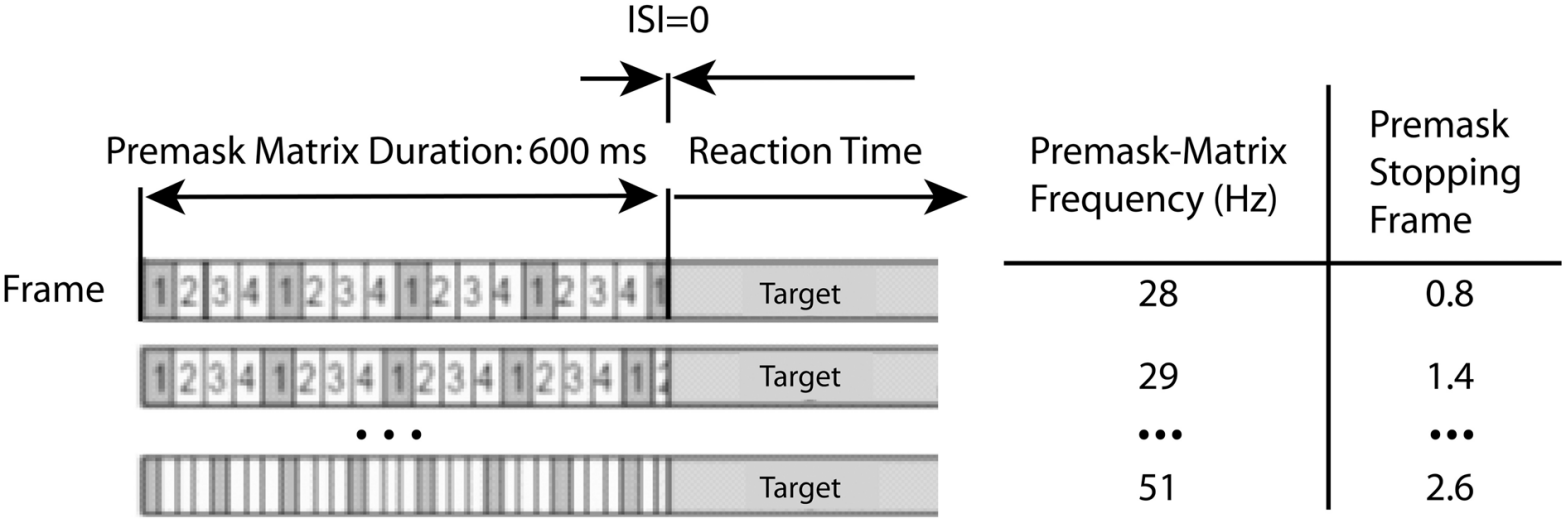
**Timing of premask-matrix—target frame presentations**. The figure illustrates the co-variation between premask-matrix frequency and the premask stopping frame that results from a fixed overall premask duration of 600 ms and variable frequencies. A premask stopping frame of 1.4, for example, denotes the situation that frame 1 is the last fully presented frame at the time of target presentation which occurs 0.4 frames through presentation (completion) of the next frame (frame 2).

For the present experiments, averaged data over premask-presentation frequency were examined using a simplified version of the procedure described by Kompass and Elliott (2001). For each frequency, the time of target presentation (600 ms post-trial onset) is transformed into a corresponding phase of the premask-matrix cycle. In Figure 5 the subtraction of the mean intra- from interphase RTs (for some frequencies the priming effects) are plotted for each frequency over the resulting phase. Figure 6 shows schematically that targets will be presented at a different phase (frame) in the cycle of premask-frame presentations dependent upon the relation between premask-matrix frequency and the premask stopping frame given a fixed overall premask duration of 600 ms. In Figure 5, the abscissa is divided into two where Phase = 0 represents the onset of frame 1—the priming frame. To the left of this are represented frequencies at which the target appears at a phase corresponding to frames 1 and 2, and to the right are frequencies at which the target appears at phases corresponding to presentation of frames 3 and 4. In this conceptualization the onsets of frames 3 and 4 are represented by negative phase values because, relative to the cyclical premask-matrix presentation, they occur at times more proximal to the subsequent presentation of priming frame 1. Positive values represent frames more proximal to a previous onset of frame 1. What is clear from inspection of Figure 5 is that the maximum differences between intra- and interphase RTs occur for targets presented at times that peak midway through the frame 4 phase and so are slightly ahead in phase relative to frame 1. In this respect, the data of Study 1 is overall consistent with the RPH described by Kompass and Elliott (2001) and indicates that a prime develops in advance of priming-stimulus presentation. The most conservative parametric estimates of the RPH derive from fitting a curve to the data (presented in Figure 5). This analysis indicates that priming should be expected to be at its maxima during the first quarter of the presentation phase of frame 4 (indicated in Figure 5). Consideration of the lowest and least frame-1 proximal of the priming frequencies contributing to the frame 4 peak (32 Hz Table 4), suggests the prime can be developed as much as 24 ms ahead of priming-stimulus presentation.

## Discussion of study 1

Experiments 1–7 show that oscillatory priming, originally found by Elliott and Müller (1998) to be particular to 40-Hz and subsequently by Elliott and Müller (2004) to occur selectively but at regularly spaced frequencies in the 28–51-Hz band, both replicates and extends as far as 67 Hz. On this basis it may be concluded that the capacity for oscillatory prime formation is present across the majority, if not all of the EEG gamma band. The GPAH is shown here to refer to a slower, presumably internal rhythm of 6.69 Hz, with which it is believed premask-matrix presentation frequencies must phase align in order to bring about the priming effects (see Figure 2).

Related to this, analysis of the RPH reveals priming to occur for target presentation times that correspond in phase with the final frame of the sequence of premask-image frames. In other words, the prime forms at a time preceding presentation of the priming frame. This leads us to ask whether the RPH is a conclusive description of the conditions required for priming. At issue is a problem of confusability between the RPH and GPAH, brought about because premask matrices were all presented for 600 ms irrespective to frequency. Four * 149 ms (the 1/*f* representation of the GPAH value of 6.69 Hz) is almost equivalent to the time of premask-matrix termination (596 vs. 600 ms). Perhaps some or all priming frequencies are favored due to the interaction of premask-matrix termination time with the slower (149 ms/6.69 Hz) rhythm?

**Figure 7:**
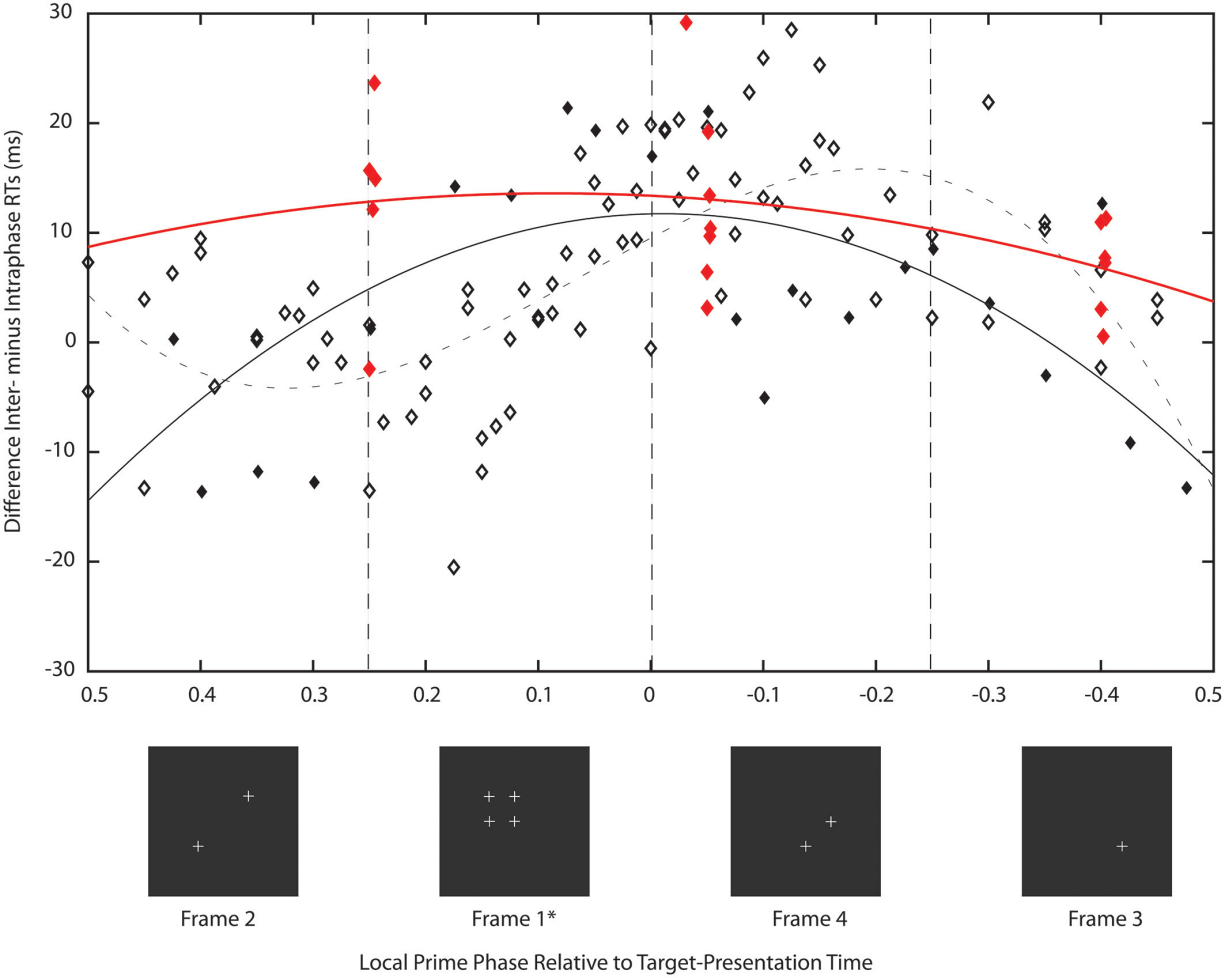
**Description of this figure is identical to that of Figure 5 except in Experiment 8 premask matrices oscillated for 700 ms and in Experiment 9, for various frequencies, presentation times corresponded to cycles of 21 (frame phase 0.25), 22.4 (phase −0.4), and 23.8 (phase −0.05) of the stimulating frequency**. Analysis of Study 1 (described in Figure 5, symbols and function here in gray) is also presented for the purpose of comparison. The priming effects recorded in Experiment 8 (black symbols and function) are unlike those of Study 1 in that almost all priming frequencies lie in the phase range 0.09 (47 Hz) to −0.02 (34 Hz) and are centered on target presentation in phase with priming stimulus (frame 1) presentation. The solid black line is a non-linear function that describes the pattern of mean intra- from interphase RTs over premask-matrix phase [*y* = −8.5^*^z^2^ − 0.37^*^z + 12, where *z* = (*x* − −0.0083)/0.58; *F*_(2, 21)_ = 8.16, *p* < 0.005] and confirms peak priming to occur at phase 0. As expected the priming effects recorded in Experiment 9 are similar to those of Experiment 8 (red symbols, rounding errors cause some phases to be slightly out of alignment) with priming in the range 0.25 to −0.05 and so primarily retroactive. The solid red line is the corresponding non-linear function that describes the pattern of mean intra- from interphase RTs over premask-matrix phase [*y* = −2^*^z^2^ + 2.6^*^z + 12, where *z* = (*x* − −0.017)/0.52, function estimated on the limited set of phase means that allow no estimate of significance]. This function suggests peak priming to occur at a phase of 0.09. Importantly and given a lack of interaction with frequency, Experiment 9 indicates return phase and not frequency as key to oscillatory priming.

There were two experiments in Study 2: experiment 8 sought clarity on the issue discussed above by using frequencies in the range 28–51 Hz in single Hertz steps while setting premask-matrix presentation time to 700 ms. A presentation time of 700 ms does not harmonically relate to the GPAH rhythm and so allows examination of the hypothesis that it is the matching phase relation that exists between priming frequencies and 6.69 Hz at 149 ms that brings about the priming effects (illustrated in Figure 2). While priming effects have been demonstrated at various premask-matrix presentation times for 40 Hz (Elliott and Müller, 1998), in each case premasks were presented at close to integer multiples of 149 ms (300, 600, 1200, 2400 ms) and a regular frequency sequence was not examined. Experiment 9 was based on two findings in Experiment 8. The first refers to the function describing priming over phase (illustrated subsequently in Figure 7). This shows maximum priming at phase 0, and so at the time when targets were presented in phase with priming-frame (frame 1) presentation. The second is the finding that priming occurs across frequency bands rightward shifted by approximately 1 Hz relative to the priming frequencies recorded in Study 1 and previous studies (Elliott and Müller, 1998, 2004). Experiment 9 tested the RPH directly by setting premask-presentation times at different premask-matrix return phases at which priming was expected to differ irrespective to premask-matrix presentation frequency. Accordingly, it also questioned the frequency-specificity of oscillatory priming.

## Study 2 materials and methods

In Experiment 8 there were 12 participants (4 male, mean age 24.1, all with normal or corrected-to-normal vision) and in Experiment 9 there were 15 participants (5 male, mean age 23.5, all with normal or corrected-to-normal vision). In Experiments 8 and 9, participants were paid at a rate of €8.00 per session. All participants provided informed consent to participate in the experiments, with protocols approved by a departmental research ethics committee convened at the Ludwig-Maximilians University, Munich.

The design and procedure used in Experiment 8 matched that of Experiment 2, except the 24 frequencies in the range 28–51 Hz were presented in a single design with 3840 trials delivered over 5 sessions with 16 blocks per session. Unlike Experiment 2, Experiment 8 employed a premask-matrix presentation time of 700 ms.

In Experiment 9, the design and procedure used matched that of previous experiments with the following exceptions. Six frequencies were presented with single-Hertz resolution in the range 35–40 Hz in a single design with 2592 trials delivered over 3 sessions with 18 blocks per session. As in Experiment 8, premask-matrix presentation time was varied but in the following manner: for each premask-matrix presentation frequency and independent of frequency, the premask matrix was presented for times equivalent to 21, 22.4, or 23.8 premask-matrix frame presentations. This varied premask-presentation time for each frequency × frame number combination is illustrated by the following examples: for premask matrices presented at *f* = 36 Hz—equivalent to 1/*f* = 27.78 ms, premask-matrix presentation times would be 583, 622, and 661 ms (corresponding to 21, 22.4, and 23.8 premask-matrix frame repeats, respectively). Reference to Figure 5 confirms that these multiples correspond to premask- presentation frame phases of 0.25 (in phase with the end of frame 1), −0.4 and −0.05. For premask matrices presented at *f* = 39 Hz—equivalent to 1/*f* = 25.64 ms, premask-matrix presentation times would be 538, 574, and 610 ms [corresponding to 21, 22.4, and 23.8 premask-matrix frame repeats and corresponding to premask- presentation frame phases of 0.25 (in phase with the end of frame 1), −0.4 and −0.05, respectively].

Given only one frequency × frame phase combination corresponds with priming as predicted by the GPAH, in Experiment 9 we expected peak priming to occur for targets presented slightly ahead and immediately after frame 0 (i.e., for 23.8 [frame phase −0.05] and 21[frame phase 0.25]) but not for targets presented following 22.4 (frame phase −0.4) premask-matrix frame presentations. In this way, Experiment 9 was a direct test of the RPH, but also examined whether particular premask-presentation frequencies are necessary for oscillatory priming.

In all other respects the methods used in Experiments 8 and 9 were identical to those described for the previous experiments.

## Results of study 2

As with the previous experiments, trials with error responses were removed from the data prior to subsequent analyses. In Experiment 8 there were 2116 errors (4.59% of all trials), and in Experiment 9 there were 1007 errors (2.59% of all trials). In both cases the error RTs tended to be slower overall than correct RTs, and analysis for speed-accuracy relations using the method recommended by Grice et al. (1977) revealed no significant correlations, arguing against the correct data being contaminated by accuracy-speed trade-offs. As with the previous experiments, repeated-measures ANOVA revealed no systematic patterns in either set of error data. Examination of the correct RTs revealed non-normal distribution with pronounced positive skew. A Kolmogorov “D” test showed RT distributions to be approximately lognormal and on this basis subsequent analyses were conducted on the exponents of the means of log-transformed RT distributions. Analysis of Experiment 8 was carried out as previously described for Study 1. For Experiment 9, a repeated measures ANOVA was carried out on the factors Target (present/absent and indicative of search), Prime (intra vs. inter and indicative of priming), *f* (premask-matrix presentation frequency) and Frame Phase (frame phases 0.25, −0.4, and −0.05, equivalent to 21, 22.4, and 23.8 premask-matrix frame repetitions).

In Experiment 8 priming overall was found to be weaker than in Experiment 2 leading to the reduced significance of the Target × Prime interaction (Table 5). What is clear is that there are still frequencies at which priming occurs selectively and that some of these frequencies differ from those in Experiment 2. In fact, there are similar bands of priming frequencies in Experiment 8 to those in Experiment 2, but these bands appear to be rightward shifted by around 1 Hz in the frequency dimension with priming occurring at slightly faster frequencies than in Study 1.

**Table 5:** **RT data ANOVA Table for Experiments 8 and 9**.

**#**	***f***	***T***	***P***	***T × P***	***P × f***	***T × P × f***
8	28–51	*F*_(1, 11)_ = 22.8; *p* = 0.001	NS	*F*_(1, 11)_ = 4.6; *p* = 0.055	*F*_(6.2, 67.8)_ = 2.8; *p* < 0.025	NS
		***T(P ×f)***	***T(Pf)***			
		*F*_(5.9, 65.2)_ = 3.2; *p* < 0.01	{28 Hz, 22 ms; *p* = 0.001} {34 Hz, 22 ms; *p* < 0.05} {40 Hz, 17 ms; *p* = 0.001} {41 Hz, 15 ms; *p* < 0.005} {46 Hz, 20 ms; *p* < 0.025} {47 Hz, 16 ms; *p* < 0.07}			
		***T***	***P***	***T × P***	***P × Ph***	***T × P × Ph***
9	35–40	*F*_(1, 14)_ = 19.6; *p* = 0.001	*F*_(1, 14)_ = 17.3; *p* = 0.001	*F*_(1, 14)_ = 6.8; *p* < 0.025	*F*_(1.3, 18.3)_ = 4.5; *p* < 0.05	NS
		***T(P × Ph)***	***T(Ph)***			
		*F*_(1.5, 20.6)_ = 3.7; *p* = 0.053	{21 15 ms; *p* = 0.001 {23.8 10 ms; *p* < 0.005}			

*T, Target; P, Prime; f, premask-matrix presentation frequency. Huynh-Feldt or Greenhouse Geisser adjustments applied where sphericity assumptions are not met. T(P × f) interactions reported here refer to the Prime × f interaction in the target trials only. T(Pf) refers to target-priming effects. For Experiment 9, T(P × PH) and T(Ph) refer to Prime × Frame Phase interactions in the target trials. There were no significant Prime × Frame Phase × Frequency interactions in Experiment 9*.

Of particular importance is the transformation shown in Figure 7. Plotted in comparison with data from Study 1 (gray symbols and function, described fully in Figure 5), the black function and symbols present data from Experiment 8. These show that priming effects no longer occur for frequencies at which the target appears at phases corresponding to presentation of frames 3 and 4, but are instead exactly centered on phase = 0, which is the time equivalent to the onset of frame 1—the priming frame. The Lomb-Scargle periodogram presented as the red function in Figure 4 shows that the separation of frequencies can no longer be described in terms of a significant regular pattern at any frequency. Experiment 8 thus shows that for premask-matrices terminating at a time out of phase with the 6.69 Hz (the rhythm characterizing the separation and therefore phase alignment of priming frequencies in Study 1), prime generation no longer appears to precede, but achieves maximum activation in synchrony with priming-stimulus presentation.

The results of Experiment 9 are consistent with the expectation that if premask-matrix presentation times vary and are not necessarily in phase with the slow theta rhythm of 6.69 Hz, prime maxima will not precede but will be aligned with or follow frame phase 0 at which target presentation aligns with frame 1 or priming-stimulus presentation. In addition and importantly, the pattern of priming effects over frame phase, alongside an absence of major interactions between Prime, Frame Phase and *f*, indicate the Prime × Frame Phase interactions do not vary as a function of premask-matrix presentation frequency. Consistent with expectations, based initially upon the RPH but guided more specifically by the results of Experiment 8, peak priming was found when targets were presented immediately following presentation of frame 1 with priming also evident at a time equivalent to the end of frame 4 (e.g., for 23.8 [frame phase −0.05] and 21[frame phase 0.25]) premask-matrix frame presentations. Priming was not found for targets presented following 22.4 (frame phase −0.4) premask-matrix frame presentations. This pattern of results clearly indicates that it is return phase and not frequency alone that determines which premask-matrix presentation frequencies prime target detection. Review of the priming frequencies supports this: the mean or greater than mean inter minus intraphase RTs were found for premask matrices presented at 35 and 39 Hz (frame phase 0.25) and 36, 37, and 38 Hz (phase −0.05). No effects were previously found for premask matrices presented at 35, 36, or 37 Hz, while Experiment 9 failed to record priming effects when premask matrices flickered at 40 Hz.

## General discussion

Study 1 shows that oscillatory primes generated through repeated and regular priming stimulus presentation, achieve maximum priming potential at a presentation phase that predates the phase of priming-stimulus presentation. This is a strong endorsement of the Return Phase Hypothesis (RPH), which also appears to model priming more reliably than either expectation that particular frequencies will prime preferentially over others (e.g., 40 Hz, Elliott and Müller, 1998, 2000), or the Generalized Phase Angle Hypothesis (GPAH) which predicts anticipatory but not synchronized or retroactive prime activation maxima.

In consideration of presentation parameters such as premask-frame presentation frequency and the phase of target presentation, this appears attributable to an interaction between premask-frame presentation frequency and an internal rhythm of 6.69 Hz. Varying premask-matrix presentation time and thereby the phase of subsequent target presentations, Study 2 appears to corroborate this conclusion. Experiments 8 and 9 show that when targets appear out of phase with the 6.69 Hz rhythm, target priming is weaker and no longer achieves its maximum at a premask-matrix presentation phase ahead of the phase of priming-stimulus presentation. On the one hand a finding of pre-activation in cognitive mechanisms coding regular stimulus events should not be surprising: it is well-known that in order to respond to movement successfully trajectories need to be computed in advance of the event so, for instance, one can place ones hand in the correct position in space at the correct time to successfully intercept and catch the incoming ball. On the other, it is unexpected that anticipatory coding occurs in conjunction with a slow oscillation in a band more commonly associated with working memory (and so in this instance retroactive coding—see Klimesch et al., 2005). This may indicate EEG theta rhythms are more closely concerned with temporal coding than previously thought, whether that coding is pre- or retroactive).

Anticipatory coding is not a new phenomenon as evidenced in tapping tasks using stimuli in isochronous visual sequences (e.g., Klemmer, 1957, 1967) referred to as Negative Mean Asynchrony, tapping slightly ahead of metronomic stimulus presentations is reviewed in Aschersleben (2002), and Repp (2005). Consideration of frequency highlights one similarity between the tapping studies and those presented here: notably and given motor and other physical constraints, the upper rate limit for 1:1 synchronization between stimulus and tapping response is observed at rates of 5–7/s and so at rates which include, and may be in synchrony with the 6.69 Hz reported here. It seems quite possible that an anticipatory response may generate in sensori-motor mechanisms following premask-matrix presentations at particular frequencies, and that this response is based upon the phase alignment of premask-frame frequency with the slow theta oscillation also measured in tapping. However, (and with it in mind that sensori-motor may refer to the eye muscles as equally as it does to those resulting in a finger movement), claiming similarities must, in the present context, also acknowledge major differences between the design of tapping studies and that of the experiments presented here. In the present experiments participants do not respond to the beat and are not required to pay attention to any temporal aspects of the premask matrix to successfully complete the detection task, particularly not the rates of premask-frame presentations which are in any case too fast to be resolved perceptually. This qualifies acceptance that the upper rate limits of tapping and the internal rhythm measured here are the same rhythm, a question resolved by combining paradigms in future research. Task differences may, however, account for why a regular anticipatory priming effect is observed across a fairly broad and very fast frequency band, whereas anticipatory tapping tends to decrease with increasing tempo and occurs at much slower frequencies.

Of major significance, the experiments presented here show that anticipatory priming (prime pre-activation) occurs maximally when priming frequencies are in phase with the 6.69-Hz rhythm, but not when frequencies and rhythm are out-of-phase. Under the latter conditions, priming occurs for targets presented at phases either synchronous with or slightly after the phase of priming-stimulus presentation. Consequently, a summary hypothesis is that anticipatory coding occurs generally given a phase alignment of the oscillation imposed by the stimulus, or the oscillation at which stimulus coding occurs spontaneously, with the cycle of a slower oscillation. It can also be concluded that anticipatory coding does not take place when this interaction does not occur. It cannot be concluded, however, that 6.69 Hz is the only slow frequency permitting this interaction; mathematically several other frequencies may align phases at several other times leading to the possibility of other slow rhythms being instrumental in anticipatory coding. Some evidence that this may not be the case derives from Experiment 8 in which similar frequencies were found to prime as in Study 1, but at a different phase, where a different series of frequencies might be expected to prime if there had been an interaction with a rhythm of 5.7 Hz (700 ms is a multiple of 5.7 Hz, which has periods of 175 ms duration). However, a more systematic investigation is required in which premask-matrix presentation time is varied while holding premask-matrix frequency constant to reach any firm conclusions about the exclusivity of 6.69 Hz.

While it is interesting that prime pre-activation seems to occur only as a function of the phase interaction between different oscillations, it is possible with some caution to relate this with other phenomena: for example, induced and evoked gamma responses in physiological and EEG recordings and Husserl's notion of protention. The prime here is a spatial grouping that expedites detection of a subsequently presented visual grouping (the target) and on this basis the paradigm of Elliott and Müller has been discussed in terms of the visual-binding literature (Gray, 1999; Singer, 1999, for reviews). In this literature oscillations are induced by stimulus activity and are not usually phase related to that activity, while in a second literature (e.g., Herrmann et al., 1999), visual binding is evoked (that is to say phase related) selectively by presentation of a grouping stimulus. In the present experiments, examples of binding (priming) are discussed in terms of oscillatory-stimulus presentations at gamma-band frequencies. Primes are found to form both in phase with and at phases not directly related to the timing of the priming stimulus. It remains to be seen whether this priming paradigm provides a single method by virtue of which it can be shown that induced and evoked gamma responses are functionally equivalent with respects to visual binding.

With respect to protention, the prime is oscillatory and maximally active ahead of the phase in premask-matrix presentation at which the priming-stimulus frame is presented. In a sense the cognition advances in phase to end up preceding the event it codes and in this respect one might claim the prime to advance into future time—in the sense intended by Husserl. This would require a closer investigation of prime evolution over time. In this respect the study of Elliott and Müller (1998) presented premask matrices for 300 ms as well as for longer durations in an unblocked design, meaning participants could not know in advance for how long a given matrix would flicker. They found no differences between frequencies in terms of priming potential, which would suggest that by 600 ms the prime is pre-active (and therefore in future time relative to priming stimulus as well as priming stimulus presentation). However, the intraphase premask that generates the prime is not detected by participants for premask-matrices presented at frequencies higher than 21 Hz (Elliott and Müller, 1998) and while visible in the overall context of the premask matrix, the contents of this frame are not distinguishable temporally from the contents other frames. This indicates that, if pre-active, the prime cannot be considered protentive in the sense intended by Husserl—that is, fully realized as a unique psychological event in consciousness, ahead of the actual event. Instead we might assume that conscious realization of events is mediated sometimes by anticipatory cognition and sometimes by cognition that occurs subsequent to the coded event. On this assumption we might conclude that if conscious experience itself is not protentive, the cognitive basis for conscious experience shifts between past, present and future times even though this cognition would not result in a relative time-stamped experience of event structure. In so far as our cognition at this level provides a content structure for consciousness, our psychological lives may be fundamentally based upon the ability of our cognitive states to travel backwards and forwards across very short intervals of time.

### Conflict of interest statement

The author declares that the research was conducted in the absence of any commercial or financial relationships that could be construed as a potential conflict of interest.
